# Threatened Poisoning of the Rhine

**Published:** 1878-05

**Authors:** 


THREATENED POISONING ON THE RHINE.—Toward the end of the past year the screw steamer "Schelde und Rhyin," having on board 656 casks of arsenic (arsenious acid), foundered in the river Rhine near Engers. Great fear was entertained lest the poisonous cargo might be penetrated by the water, and destroy the lives of the animals and of inhabitants along the river below the place of the accident. The boat and cargo were raised with as little delay as possible, and only five of the casks were found to be damaged by water, none of them having lost any considerable portion of arsenic. The consequence of the collapse of all the casks and the mixture of their contents with the river would have been most disastrous. --*The Clinic.*

